# Transcriptomic Stress Response in *Streptococcus mutans* following Treatment with a Sublethal Concentration of Chlorhexidine Digluconate

**DOI:** 10.3390/microorganisms10030561

**Published:** 2022-03-04

**Authors:** Denise Muehler, Xiaojun Mao, Stefan Czemmel, Janina Geißert, Christina Engesser, Karl-Anton Hiller, Matthias Widbiller, Tim Maisch, Wolfgang Buchalla, Ali Al-Ahmad, Fabian Cieplik

**Affiliations:** 1 Department of Conservative Dentistry and Periodontology, University Hospital Regensburg, 93053 Regensburg, Germany; denise.muehler@ukr.de (D.M.); olivermxj@sina.com (X.M.); karl-anton.hiller@ukr.de (K.-A.H.); matthias.widbiller@ukr.de (M.W.); wolfgang.buchalla@ukr.de (W.B.); 2 Quantitative Biology Center, University of Tübingen, 72074 Tübingen, Germany; stefan.czemmel@qbic.uni-tuebingen.de; 3 NGS-Competence Center Tübingen, Institute for Medical Microbiology and Hygiene, University Hospital Tübingen, 72076 Tübingen, Germany; janina.geissert@uni-tuebingen.de (J.G.); christina.engesser@uni-tuebingen.de (C.E.); 4 Department of Dermatology, University Hospital Regensburg, 93053 Regensburg, Germany; tim.maisch@ukr.de; 5 Department of Operative Dentistry and Periodontology, Faculty of Medicine, University Freiburg, 79085 Freiburg, Germany; ali.al-ahmad@uniklinik-freiburg.de

**Keywords:** antibacterial, CHX, RNA-seq, *Streptococcus mutans*, stress response, transcriptomics

## Abstract

Despite the widespread use of antiseptics such as chlorhexidine digluconate (CHX) in dental practice and oral care, the risks of potential resistance toward these antimicrobial compounds in oral bacteria have only been highlighted very recently. Since the molecular mechanisms behind antiseptic resistance or adaptation are not entirely clear and the bacterial stress response has not been investigated systematically so far, the aim of the present study was to investigate the transcriptomic stress response in *Streptococcus mutans* after treatment with CHX using RNA sequencing (RNA-seq). Planktonic cultures of stationary-phase *S. mutans* were treated with a sublethal dose of CHX (125 µg/mL) for 5 min. After treatment, RNA was extracted, and RNA-seq was performed on an Illumina NextSeq 500. Differentially expressed genes were analyzed and validated by qRT-PCR. Analysis of differential gene expression following pathway analysis revealed a considerable number of genes and pathways significantly up- or downregulated in *S. mutans* after sublethal treatment with CHX. In summary, the expression of 404 genes was upregulated, and that of 271 genes was downregulated after sublethal CHX treatment. Analysis of differentially expressed genes and significantly regulated pathways showed regulation of genes involved in purine nucleotide synthesis, biofilm formation, transport systems and stress responses. In conclusion, the results show a transcriptomic stress response in *S. mutans* upon exposure to CHX and offer insight into potential mechanisms that may result in development of resistances.

## 1. Introduction

Despite the current COVID-19 pandemic, antimicrobial resistance (AMR) remains one of the greatest challenges for public health in the 21st century [1,2]. For instance, a 2018 report of the Organization for Economic Co-operation and Development (OECD) predicted that 2.4 million people in Europe, North America and Australia will die from infections associated with AMR within the next 30 years, resulting in costs of up to USD 3.5 billion per year for the healthcare services of the 33 countries included in the analysis [3]. Despite many reports about antibiotic resistance, antiseptics or biocides have only recently come into spotlight in the context of AMR [4,5,6,7].

Chlorhexidine is a twofold positively charged bis-biguanide and is mostly used as its digluconate salt (chlorhexidine digluconate, CHX) in clinical practice [4,8]. Since its introduction into dentistry in the late 1960s [9], CHX has come to be considered the gold-standard antiseptic in oral care [4,10]. It is used for plaque control and management of gingivitis [11]; in patients with high caries risk, such as those with fixed orthodontic appliances [12]; or in patients after periodontal or implant surgery [13]. Furthermore, CHX is included in a wide range of over-the-counter oral care products, such as toothpastes or mouthwashes [11,14,15].

Although there is ample evidence that rinsing with CHX can decrease the salivary bacterial load [16] and consequently reduce oral biofilm formation [17,18], the antibacterial efficacy of CHX toward mature oral biofilms may be limited [4,19,20]. For instance, we recently showed that microcosm biofilms from human saliva cultured for 72 h and treated with 0.1% or 0.2% CHX for 1 min resulted in colony-forming-unit reduction rates of less than 1 log_10_ step [20]. Accordingly, treatment with 0.2% CHX for 1 min only affected the outer layers of biofilms formed in situ for 48 h [19]. This limited efficacy of CHX in mature oral biofilms may be mainly due to the biofilm matrix, which acts as a diffusion barrier for positively charged molecules, such as CHX [21]. Consequently, it is reasonable that bacteria in deeper layers will be exposed to sublethal concentrations of CHX upon application of a CHX-containing mouthwash [4,20]. Several studies from recent years showed that oral, as well as non-oral, bacteria can phenotypically adapt upon multiple exposure to sublethal concentrations in vitro [20,22,23,24,25] and potentially also develop cross resistances toward antibiotics [23,25]. However, the actual molecular mechanisms causing these adaptations, as well as the stress response in bacteria upon exposure toward sublethal concentrations of CHX, are not well understood yet [15].

Therefore, the aim of the present study was to investigate the transcriptomic stress response after sublethal treatment with CHX using an RNA sequencing approach with *Streptococcus mutans* as model organism due to its key role in biofilm formation and pathogenesis of dental caries [26,27].

## 2. Materials and Methods

### 2.1. Chlorhexidine Digluconate

Chlorhexidine digluconate (CHX) was obtained from Sigma-Aldrich (Merck, Darmstadt, Germany) in a concentration of 20% and was dissolved in distilled water to yield the final concentration of 125 µg/mL (0.0125%).

### 2.2. Bacterial Culture and Treatment

A-type strain of *S. mutans* (DSM 20523; ATCC 25175) was obtained from the German Collection of Microorganisms and Cell Cultures GmbH (DSMZ; Braunschweig, Germany) to be used in this study. *S. mutans* was grown in brain heart infusion broth (BHI; Sigma-Aldrich, St. Louis, MO, USA) and on BHI agar plates. Planktonic cultures (5 mL) were grown aerobically overnight at 37 °C. Afterwards, suspensions were harvested by centrifugation (ROTINA 420 R, Hettich Lab Technology, Tuttlingen, Germany) and resuspended in BHI, yielding an optical density (OD) of 2.0 per mL, as measured by means of a spectrophotometer at 600 nm (Ultrospec 3300 pro; Amersham Biosciences, Amersham, UK). Samples were centrifuged, supernatants were discarded and bacterial pellets were resuspended and incubated with 200 μL BHI broth or 200 µL CHX (final concentration: 125 µg/mL) for 5 min.

To evaluate the bacterial ability to replicate, samples were centrifuged, BHI broth or CHX solution was carefully removed, and the bacterial pellet was brought to suspension with 200 μL phosphate-buffered saline (PBS; Biochrom, Berlin, Germany) by frequent pipetting. Tenfold serial dilutions (10^−2^ to 10^−7^) were prepared in PBS, and aliquots (20 μL) were plated on BHI agar (both provided by the Institute of Medical Microbiology and Hygiene, University Hospital Regensburg, Germany) according to the method described by Miles et al. [28]. Samples were incubated aerobically for 72 h, and subsequently colony forming units (CFUs) were evaluated (*n* = 5).

For RNA-Seq, samples were centrifuged after treatment, and distilled water or CHX solution was carefully removed. Pellets were resuspended in 500 μL prewarmed BHI broth and incubated at 37 °C for 30 min. Afterwards, 1 mL of RNAprotect bacteria reagent (Qiagen, Hilden, Germany) was added to the samples and incubated for 5 min at room temperature. Then, samples were centrifuged at 17,035× *g* for 5 min, supernatants were discarded and pellets were stored at −80 °C until further use (*n* = 5).

### 2.3. RNA Extraction, Library Preparation and RNA Sequencing

RNA extraction and sequencing were performed by the NGS Competence Center Tübingen (NCCT), the sequencing partner of the Quantitative Biology Center (QBiC), Tübingen, Germany. RNA of all samples was isolated with the Quick-RNA fungal/bacterial miniprep kit (Zymo Research, Freiburg, Germany). RNA extraction was followed by DNase I treatment in solution using the DNase I recombinant kit (Roche, Basel, Switzerland), as well as purification and concentration of RNA using the RNA Clean & Concentrator-5 kit (Zymo Research, Freiburg, Germany).

Library preparation was performed using an Illumina Stranded Total RNA Prep Ligation with the Ribo-Zero Plus kit (Illumina, San Diego, CA, USA) with 100 ng total RNA. Sequencing was performed on an Illumina NextSeq 500 using a high-output kit (version 2.5; 75 cycles) with 1% PhiX spike-in (Illumina, San Diego, CA, USA).

### 2.4. RNA Sequencing Data Analysis

Data management and bioinformatic analysis were performed at the Quantitative Biology Center (QBiC), Tübingen, Germany. A Nextflow-based nf-core pipeline nf-core/rnaseq (version 1.4.2; https://github.com/nf-core/rnaseq, accessed on 22 January 2022) was used for the RNA-seq bioinformatic analysis. As part of this workflow, FastQC (version v0.11.8) was used to determine the quality of the FASTQ files [29]. Subsequently, adapter trimming was conducted with Trim Galore (version 0.6.4) [30]. STAR aligner (version 2.6.1, [31]) was used to map the reads that passed quality control against the latest assembly (RefSeq assembly accession GCF_000347875.1) of strain ATCC 25175, downloaded from NCBI. Note that the gtf file for the *S. mutans* genome had to be modified to be used for the nf-core/rnaseq workflow by removing empty fields for the tag “transcript_id” in the ninth column of that gtf file. RNA-seq data quality control was performed with RSeQC (version 3.0.1) [32] and read counting of the features (e.g., genes) with featureCounts (version 1.6.4) [33]. An aggregation of the quality control for the RNA-seq analysis was performed with MultiQC (version 1.7; http://multiqc.info/, accessed on 22 January 2022) [34].

The analysis of the differential gene expression was performed in R language (version 3.5.1) using DESeq2 (version 1.22) through the Nextflow-based workflow qbic-pipelines/rnadeseq (https://github.com/qbic-pipelines/rnadeseq, accessed on 22 January 2022, version 1.3.2). Genes were considered differentially expressed (DE) when the Benjamini–Hochberg multiple testing adjusted *p*-value [35] was smaller than 0.05 (*p*_adj_ ≤ 0.05). Multiple testing correction helps to reduce the number of false positives (not real DE genes). In the case of a threshold of 0.05, the proportion of false discoveries in the selected group of DE genes is controlled to be less than the threshold value—in this case, 5%. Genes were further filtered for biological relevance if the log_2_ fold change (FC) in expression between the two considered groups was above the threshold of 1.0 and less than −1.0. Final reports were produced using the R package rmarkdown (version 2.1) with the knitr (version 1.28) and DT (version 0.13) R packages. The sample similarity heatmaps were created using the edgeR (version 3.26.5) R package. BioCyc database was used for pathway analysis [36]. All DE genes were included, and enrichment was calculated using Fisher’s exact test (*p* ≤ 0.05).

### 2.5. Primer Design and qRT-PCR

To validate RNA-Seq data, randomly selected genes were measured by qRT-PCR. The nucleotide sequence of each gene (Table 1) was downloaded from BioCyc [36]. Primers were designed using the Primer3 tool (version 4.1.0, Whitehead Institute for Biomedical Research, Cambridge, MA, USA) and synthesized by Eurofins MWG Synthesis (Ebersberg, Germany). Primer efficiencies were checked by qRT-PCR using different cDNA dilutions. Primers gave a single PCR product, which was verified by gel electrophoresis and melt curves at the end of each run. Total RNA (200 ng) used for RNA-Seq was reverse-transcribed with the Quantitect reverse transcriptase kit (Qiagen, Hilden, Germany) in a reaction volume of 20 μL. Polymerase chain reaction was carried out with 20 pmol of each primer, 5 µL cDNA (0.2 ng) diluted and qPCR master mix with SYBR^®^Green in a final volume of 20 µL (Applied Biosystems^TM^ SYBR^®^Select Master Mix, Thermo Fisher Scientific, Waltham, MA, USA) on the QuantStudio 3 real-time PCR system (Thermo Fisher Scientific, Waltham, MA, USA) following an initial denaturation of the samples at 95 °C for 10 min and 40 cycles of alternating denaturation (95 °C for 1 s), annealing and elongation (60 °C for 20 s).

Gene expression was quantified by the comparative threshold cycle (Ct) method (2^−∆∆Ct^) [37]. First, Ct values of all expressed genes were normalized by the housekeeping gene *gyrB*. Furthermore, gene expression of treated cells was calculated relative to the untreated control cells (log_2_ fold change). Median values and quantiles of all experiments were calculated and compared to log_2_ fold changes obtained from RNA-seq (*n* = 5).

## 3. Results

### 3.1. Sublethal Concentration of CHX

The bacterial ability to replicate was investigated for planktonic cultures of *S. mutans* after treatment with 125 µg/mL CHX by a CFU assay. The chosen concentration of 125 µg/mL was determined by experiments screening multiple concentrations of CHX (data not shown). For the purpose of this study, sublethal doses of CHX were defined as the treatment that resulted in a < 0.5 log_10_ step reduction in CFU. Untreated controls exhibited 1.0 × 10^7^ to 1.4 × 10^7^ CFU. Samples treated with 125 µg/mL CHX for 5 min showed 4.5 × 10^6^ to 8.5 × 10^6^ CFU, resulting in a reduction in CFU of 0.2 log_10_ steps, as compared to the untreated control.

### 3.2. RNA-Seq and Analysis of Differentially Expressed Genes

**Figure 1:**
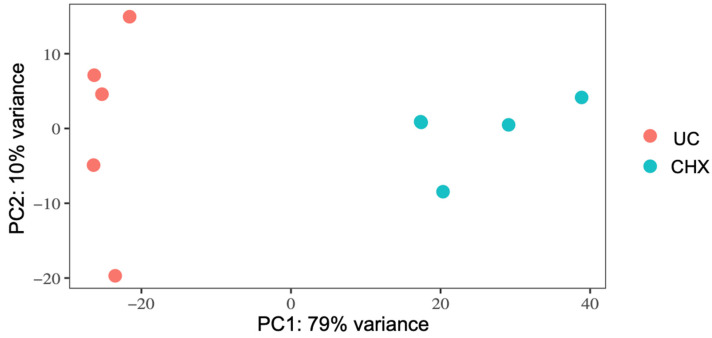
Principal component analysis (PCA) of gene expression in *S. mutans* untreated (UC, orange) or treated with CHX (turquoise). The plot shows the first two principal components (PC1 and PC2), which account for 79% and 10% of the total variation of the data, respectively. UC: untreated control.

**Figure 2:**
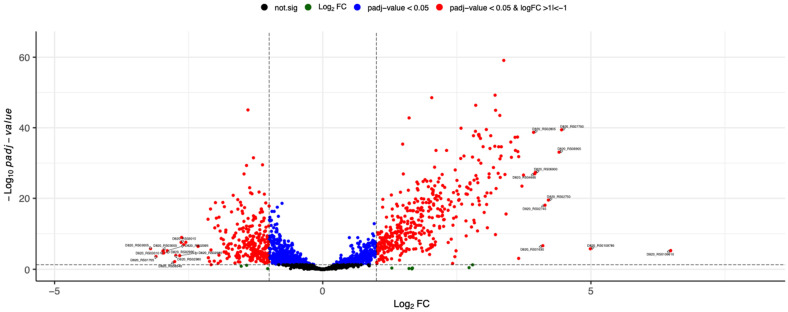
Volcano plot of differential gene expression of *S. mutans* treated with CHX versus untreated bacteria. Each point represents the average value of one transcript in five replicate experiments. The expression difference is considered significant for a multiple (FDR-based) adjusted *p*-value of 0.05 (light grey broken horizontal line). The list of DE genes was then further filtered for biological relevance by filtering on a log_2_ fold change of ≥1 and ≤−1 (red points, outer blue broken vertical lines). The top 20 DE genes based on log_2_ fold change are labelled.

RNA-seq was performed to investigate the molecular stress response of *S. mutans* upon sublethal treatment with CHX. Five independent experiments were performed on CHX-treated and -untreated bacteria. Principal component analysis (PCA) indicated that all biological replicates grouped together, suggesting that gene expression was significantly influenced by the treatment with CHX compared to untreated samples, explaining 89% of the variance observed in the complete dataset (Figure 1). The distribution of gene expression between the untreated and treated samples is represented by a volcano plot (Figure 2). The genes located outside the borders of the line fold change ≥ 1.0 were considered DE genes. The number of DE genes with an adjusted *p*-value ≤ 0.05 was 718. Genes situated on the left boundary are downregulated, whereas those on the right are upregulated genes. As compared to the untreated control, 404 genes were upregulated and 271 genes were downregulated in *S. mutans* after treatment with 125 µg/mL CHX for 5 min. A list of all genes detected by RNA-seq can be found in the Supplementary Material (Table S1).

Table 2 and Figure 2 show the 10 most up- and 10 most downregulated genes. DE genes were selected by their log_2_ fold change. It was observed that CHX treatment significantly regulated expression of genes involved in transport systems (ABC transporter and PTS system). Table 3 shows selected DE genes involved in oxidative stress (*ahpC*, *ahpF*, *sod*, *trxP* and *tpx*), general stress (*groEL, groES* and D820_RS07050) and acid stress response (*aguA* and *mleP*), which were significantly upregulated.

### 3.3. Pathway Enrichment Analysis with Differentially Expressed Genes

Pathway enrichment analysis was performed using the BioCyc database, including all upregulated DE genes or all downregulated DE genes after CHX treatment. Significantly enriched pathways that were upregulated were composed of genes involved in carboxylate degradation, glycan biosynthesis, purine nucleotide biosynthesis, L-histidine biosynthesis and L-ascorbate degradation (Table 4). Lactose and galactose degradation, protein modification and L-phenylalanine biosynthesis were found to be downregulated after CHX treatment (Table 4).

### 3.4. Validation of RNA-Seq Data Using qRT-PCR

To validate RNA-Seq data, randomly selected genes were measured by qRT-PCR to evaluate the transcription levels. Overall, the expression levels of all selected genes were similar to those of the RNA-seq, with a Spearman correlation coefficient of 0.8. The results showed that the D820_RS03003 expression levels were downregulated in CHX-treated bacteria compared to the untreated control (Table 5). In comparison, expression levels were upregulated in D820_RS09005, *ssrA* and *glgA* in CHX-treated bacteria compared to the untreated control (Table 5), which is in line with the DE results from the RNA-Seq experiment.

## 4. Discussion

Despite the widespread use of antiseptics such as CHX in dental practice and oral care, the risks of potential resistance toward these antimicrobial compounds in oral bacteria have only been highlighted very recently [4,5,20,22,24]. Since the molecular mechanisms behind antiseptic resistance or adaptation are not entirely clear yet and the bacterial stress response upon sublethal exposure toward CHX has not been investigated systematically so far [4], the aim of the present study was to investigate the transcriptomic stress response in *S. mutans* after sublethal treatment with CHX.

*S. mutans* was chosen as a model organism for the present study due to its key role with regard to biofilm formation and pathogenesis of dental caries [26,27]. A type strain was used in order to ensure that the transcriptomic stress response of a sublethal treatment with CHX was investigated on a strain not pre-adapted to CHX. For this purpose, it was necessary to choose “sublethal” treatment stress conditions but not kill the bacteria in order to allow for transcriptomic adaptations. Thus, sublethal conditions were defined as conditions that result in a reduction in bacterial ability to replicate in <0.5 log_10_ step as measured by CFU, which means that less than 50% of the bacteria are influenced in their ability to replicate. Bacteria were treated with CHX at a concentration of 125 µg/mL for 5 min, which resulted in a CFU reduction of 0.2 log_10_ steps. After treatment, bacteria were cultured in fresh nutrient broth for 30 min to allow for regulation of transcriptomic stress response. Afterwards, RNA was extracted and used for RNA-seq. RNA-seq data were validated using qRT-PCR to evaluate the transcription levels of randomly selected genes.

**Figure 3:**
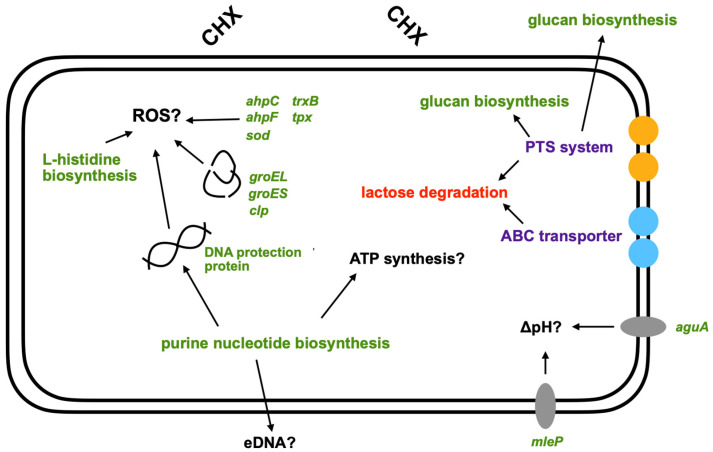
Schematic representation of a possible stress response in *S. mutans* to CHX treatment found in this study. Shown are the mechanisms described in the discussion in response to CHX treatment. Green: upregulated; red: downregulated; purple: up- and downregulated. Black arrows represent the links between different mechanisms. ROS: reactive oxygen species; PTS: phosphotransferase system; ABC: ATP-binding cassette; eDNA: extracellular DNA.

Sublethal treatment of *S. mutans* with CHX showed a strong treatment effect that led to a clear separation of samples into the two experimental groups, thereby explaining 89% of the variance in the dataset. The reason for this is most likely the differential regulation of gene expression, with 404 genes upregulated and 271 genes downregulated. Further analysis of DE genes and significantly regulated pathways showed regulation of genes involved in purine nucleotide synthesis, biofilm formation, transport systems and stress responses (Figure 3).

Amongst the DE genes and significantly regulated pathways, an upregulation in the expression of genes involved in purine nucleotide synthesis was detected. For the bacterial cell, purines primarily contribute to cell division and synthesis of necessary energy and cofactors [38]. For biofilm formation, purines are required for the production of extracellular DNA [39,40], as well as synthesis of secondary messengers from guanosine monophosphate (GMP) and adenosine monophosphate (AMP), which also play a role in the overall stress response [41]. Purine nucleotide biosynthesis has been shown to be the most regulated pathway in biofilm formation in Gram-positive bacteria [42]. Further studies have found a link between increased purine nucleotide biosynthesis and the formation of “persister” bacteria and the emergence of antibiotic resistance [43,44]. This is explained by the fact that the increase in purines leads to an increase in ATP concentration, which is needed for a stronger formation of polymers, such as for the construction of the peptidoglycan layer [43].

After treatment with CHX, regulation of ATP-binding cassette (ABC) transporters occurred. These are transport systems responsible for the uptake of nutrients, amino acids, ions or peptides, as well as the release of hydrophobic substances or toxins [45]. In addition, ABC transporters play a role in DNA repair and translation of mRNA [46,47]. Regulation of ABC transporters upon exposure to CHX has been discussed and linked to resistance formation in other studies [48,49,50,51]. Treatment of *Acinetobacter baumannii* with CHX resulted in the upregulation of the efflux system AdeAB and AceI, which were responsible for increased tolerances [50]. A study by Liu et. al. also demonstrated that deletion of the ABC transporter LmrB resulted in enhanced biofilm formation and a higher resistance to acid, H_2_O_2_, CHX, penicillin and erythromycin [52]. Another group of transporters constitutes the phosphotransferase system (PTS), members of which were also significantly regulated in *S. mutans* after treatment with CHX in the present work. The role of these transporters is mainly the uptake and phosphorylation of sugar derivatives [53,54]. Accordingly, regulation of PTS may be responsible for reduced lactose and galactose degradation. In addition, it may be related to the upregulation of glycan synthesis [55,56]. The accumulation of intracellular polysaccharides (IPS) can be enhanced by nutrient deficiency, thus helping the cell to survive by promoting the emergence of “persister” bacteria [57]. Glucan synthesis outside the cell indicates formation of extracellular polymeric substances (EPS), hinting to increased biofilm formation [58,59]. The increased biofilm formation after treatment of *S. mutans* with CHX was also demonstrated in a study by Dong et al. [60].

Sublethal CHX treatment also resulted in increased expression of specific genes encoding for the synthesis of enzymes, such as superoxide dismutase, thioredoxin, peroxiredoxin, and alkyl hydroperoxide reductases, indicating intracellular oxidative stress [61]. Oxidative stress may lead to damage of proteins or DNA [62,63]. This is confirmed by the regulation of genes encoding for chaperones (*groEL* and *groES*) and a DNA protective protein after treatment with CHX. In addition, increased expression of Clp proteases was observed, which play a major role in cell survival after stressful events, such as heat shock, acid stress or oxidative stress [64,65]. In a study by Deng et al., it was shown that deletion of the protease ClpP in *S. mutans* leads to a higher sensitivity to CHX [66]. In addition, measurement of metabolic activity showed increased tolerance to toxic H_2_O_2_ and CHX after pre-incubation with sublethal levels of the corresponding compounds in the wild-type strain but not in the mutant [66]. Accordingly, the increase in regulation of Clp proteases observed in the present work may be related to oxidative stress.

In addition to the suggestion that the regulatory changes in gene expression occur due to oxidative stress, there is also evidence of intracellular acid stress in *S. mutans* after treatment with CHX, which may be explained by the acidic pH of CHX (pH 5.5). This assumption can be supported by a study of Svensäter et al. which showed that exposure of planktonic growing *S. mutans* to pH values between 6.0 and 3.5 results in the induction of an acid tolerance response [67]. To protect themselves from low pH, oral bacteria attempt to maintain intracellular pH by alkalinization [68]. In the present work, malate permease (*mleP*) was found to be upregulated, which plays a role in malolactic fermentation. This involves the conversion of L-malate to L-lactate with the acquisition of ATP and CO_2_, leading to alkalinization of the environment [69,70]. Furthermore, an increased expression of agmatine deiminase (*aguA*) was found in this study. Agmatine is a decarboxylated derivative of arginine that is taken up by the cell and degraded by agmatine deiminase. The degradation produces ATP, CO_2_ and NH_3_, which eventually leads to an increase in intracellular pH [71,72].

## 5. Conclusions

In conclusion, we have identified and analyzed a considerable number of genes and pathways significantly regulated in *S. mutans* after sublethal treatment with CHX (Figure 3). RNA-seq showed increased expression of genes related to oxidative stress. Furthermore, regulation of transporters and increased biofilm formation were found, which could support the risk of development of resistance. Due to the acidic pH of CHX, acid stress, in addition to oxidative stress, occurred. This is the first attempt to assess the transcriptomic stress response following sublethal treatment of *S. mutans* with CHX. Further investigations should focus on the transcriptomic stress response in clinical isolates or strains exhibiting phenotypic adaptation toward CHX in order to assess potential resistance mechanisms. Furthermore, whereas the present study investigated the transcriptomic stress response in planktonic bacteria, future studies should include bacteria grown in biofilms, which may exhibit different stress responses.

## Figures and Tables

**Table 1:** Primer sequences for qRT-PCR.

Target Gene	Sequence 5′-3′ (F = Forward; R = Reverse)	Product Size
*gyrB*	F: GCACAAGAGTACGATGCCAGT	119 bp
	R: TCCCAAACAAGGTGATGCAGC	
D820_RS03005	F: CGTGGTTATCAAGTATCGTGTGA	148 bp
	R: AAAGAATTGGTCCTGCATCCA	
D820_RS09005	F: CAGTAGGTGCCGCTCAAACT	128 bp
	R: AAGTCCGCCGCCAAACATAT	
*glgA*	F: GTGCCTTGCCCAAATCCCTT	145 bp
	R: ACATATTCACGACGCCAGCC	
*ssrS*	F: CGGAAGCAACTAAAGTCAGAGCG	80 bp
	R: TGGCACCGATGATTCACGTT	

**Table 2:** The 10 most up- and downregulated genes in *S. mutans* upon sublethal CHX treatment. The full list of DE genes can be found in the Supplemental Table S1.

Gene ID	Gene Name	Product	Log_2_ FC	*p*_adj_
D820_RS03005	NA	PTS fructose transporter subunit IIB	−3.2	1.60 × 10^−6^
D820_RS01705	NA	tRNA-Thr	−3.1	2.46 × 10^−4^
D820_RS03010	NA	PTS mannitol/fructose, IIC component	−3.0	2.26 × 10^−5^
D820_RS03000	NA	PTS mannitol transporter subunit IIB	−3.0	6.57 × 10^−6^
D820_RS02990	*lacC*	Tagatose-6-phosphate kinase	−3.0	2.40 × 10^−5^
D820_RS02995	*lacD*	Tagatose-bisphosphate aldolase	−2.9	6.31 × 10^−6^
D820_RS08540	NA	tRNA-Ser	−2.8	6.90 × 10^−3^
D820_RS02980	*lacA*	Galactose-6-phosphate isomerase	−2.7	1.24 × 10^−4^
D820_RS02985	*lacB*	Galactose-6-phosphate isomerase	−2.7	1.43 × 10^−4^
D820_RS03015	NA	Lactose-specific phosphotransferase enzyme IIA component	−2.6	2.85 × 10^−8^
D820_RS02805	*glgA*	Glycogen synthase	3.9	0.00
D820_RS04685	NA	ABC transporter (ATP-binding protein)	4.0	0.00
D820_RS06900	NA	Hypothetical protein	4.0	0.00
D820_RS01630	NA	16S ribosomal RNA	4.1	2.44 × 10^−7^
D820_RS02745	NA	ABC transporter (ATP-binding protein)	4.1	0.00
D820_RS02750	NA	ABC transporter permease	4.2	0.00
D820_RS06905	*lrgB*	Antiholin	4.4	0.00
D820_RS07700	*pflB*	Formate C-acetyltransferase	4.5	0.00
D820_RS0109785	NA	23S ribosomal RNA	5.0	1.79 × 10^−6^
D820_RS0109610	NA	23S ribosomal RNA	6.5	5.73 × 10^−6^

FC: fold change; *p*_adj_: adjusted *p*-value; NA: not applicable.

**Table 3:** Differentially expressed genes in *S. mutans* upon CHX treatment related to stress response.

Gene ID	Gene Name	Product	Log_2_ FC	*p*_adj_
D820_RS06120	*ahpC*	Peroxiredoxin	1.1	1.50 × 10^−6^
D820_RS06115	*ahpF*	Alkyl hydroperoxide reductase subunit F	1.8	6.00 × 10^−17^
D820_RS06685	*sod*	Superoxide dismutase	1.6	1.71 × 10^−6^
D820_RS07435	*trxB*	Thioredoxin disulfide reductase	1.1	5.15 × 10^−4^
D820_RS05415	*tpx*	2-Cys-peroxiredoxine	1.0	1.02 × 10^−2^
D820_RS07050	NA	DNA protection protein	1.9	2.41 × 10^−12^
D820_RS00990	*groES*	Co-Chaperone GroES	1.1	8.15 × 10^−6^
D820_RS00995	*groEL*	Chaperone GroEL	1.5	0.00
D820_RS05270	NA	ATP-dependent Clp protease ATP-binding subunit	1.1	1.04 × 10^−7^
D820_RS03310	*clpB*	Clp proteinase ATP-binding subunit ClpB	2.4	4.29 × 10^−15^
D820_RS06955	NA	ATP-dependent Clp protease ATP-binding subunit	1.4	3.43 × 10^−10^
D820_RS08310	*aguA*	Agmatine deiminase	1.3	1.27 × 10^−14^
D820_RS08960	*mleP*	Malate permease	3.7	0.00

FC: fold change; *p*_adj_: adjusted *p*-value; NA: not applicable.

**Table 4:** Significantly up- and downregulated pathways in *S. mutans* upon CHX treatment.

	Pathway	Genes Involved in Pathway (Ensemble IDs)	*p*-Value
**Upregulated**			
	Carboxylate degradation	D820_RS00885, D820_RS07700, D820_RS07305, D820_RS08215, D820_RS08265, D820_RS08275, D820_RS08270, D820_RS04975, D820_RS04980, D820_RS08965	5.00 × 10^−5^
	5-Aminoimidazole ribonucleotide biosynthesis	D820_RS09340, D820_RS09355, D820_RS09335, D820_RS09300, D820_RS09345	2.00 × 10^−3^
	Glycan pathway	D820_RS07040, D820_RS02810, D820_RS02685, D820_RS02800, D820_RS02795, D820_RS02790, D820_RS02805	3.00 × 10^−3^
	L-ascorbate degradation	D820_RS08215, D820_RS08265, D820_RS08275, D820_RS08270	6.00 × 10^−3^
	L-histidine biosynthesis	D820_RS03965, D820_RS03970, D820_RS03955, D820_RS03950, D820_RS03960, D820_RS03940, D820_RS03930	7.00 × 10^−3^
	Purine nucleotide biosynthesis	D820_RS04105, D820_RS04110, D820_RS04100, D820_RS09285, D820_RS09290, D820_RS09325, D820_RS09250, D820_RS08295, D820_RS09340, D820_RS09355, D820_RS09335, D820_RS09300, D820_RS09345	1.00 × 10^−2^
	Inosine-5’-phosphate biosynthesis	D820_RS09285, D820_RS09290, D820_RS09325, D820_RS09250	2.00 × 10^−2^
**Downregulated**			
	Lactose degradation	D820_RS02995, D820_RS02980, D820_RS02985, D820_RS03025, D820_RS02990	4.00 × 10^−6^
	Galactose degradation	D820_RS02995, D820_RS02980, D820_RS02985, D820_RS03025, D820_RS02990	4.00 × 10^−6^
	Protein modification	D820_RS05715, D820_RS02255, D820_RS08010	2.00 × 10^−2^
	L-phenylalanine biosynthesis	D820_RS03740, D820_RS07095	2.00 × 10^−2^

**Table 5:** Validation of differentially expressed genes using qRT-PCR. Transcript levels of selected genes (Table 1) were corrected to *gyrB*. Each value (log_2_ fold change) is the median of five replicate PCR reactions.

Gene	RNA-Seq	qRT-PCR
D820_RS03005	−3.2	−2.4
D820_RS09005	3.3	3.4
*ssrS*	3.4	2.5
*glgA*	3.9	4.0

## Data Availability

All data supporting the reported results are available upon request from the corresponding author. The RNA-seq data discussed in this publication have been deposited in NCBI’s Gene Expression Omnibus [73] and are accessible through GEO Series accession number GSE197633 (https://www.ncbi.nlm.nih.gov/geo/query/acc.cgi?acc=GSE197633, accessed on 22 January 2022).

## Supplementary Materials

The following supporting information can be downloaded at: https://www.mdpi.com/article/10.3390/microorganisms10030561/s1, Table S1: List of all genes of *S. mutans* detected by RNA-Seq.
